# Evolvement of Peer Support Workers’ Roles in Psychiatric Hospitals: A Longitudinal Qualitative Observation Study

**DOI:** 10.1007/s10597-020-00741-1

**Published:** 2020-12-24

**Authors:** Alexa Nossek, Anna Werning, Ina Otte, Jochen Vollmann, Georg Juckel, Jakov Gather

**Affiliations:** 1 Institute for Medical Ethics and History of Medicine, Ruhr University Bochum, Markstraße 258a, 44799 Bochum, Germany; 2 Department of Psychiatry, Psychotherapy and Preventive Medicine, LWL University Hospital Bochum, Ruhr University Bochum, Alexandrinenstraße 1-3, 44791 Bochum, Germany; 3 LWL Institute of Mental Health, LWL University Hospital Bochum, Alexandrinenstraße 1-3, 44791 Bochum, Germany

**Keywords:** Experienced involvement, Adult psychiatry, Mental healthcare, Implementation, Qualitative empirical research

## Abstract

Peer support workers (PSWs) use their experiential knowledge and specific skills to support patients in their recovery process. The aim of our study was to examine the integration and role-finding process of PSWs in adult psychiatric hospitals in Germany. We conducted open nonparticipant observations of 25 multiprofessional team meetings and 5 transregional peer support worker meetings over a period of six months. The data were analyzed using qualitative content analysis. Regarding the integration of PSWs into multiprofessional teams, we identified three subcategories: “Features of success,” “challenges” and “positioning between team and patients.” Concerning the PSWs’ roles, we developed two subcategories: “Offers” and “self-perception.” The PSWs’ specific roles within a multiprofessional mental healthcare team evolve in a process over a longer period of time. This role-finding process should be supported by a framework role description which leaves sufficient freedom for individual development. Regular opportunities for mutual exchange among PSWs can help to address specific support needs at different points in time.

## Introduction

The concept of peer support work in mental healthcare means the involvement of people with lived experience in the treatment of people with mental health challenges. Peer support workers (PSWs) aim at promoting hope by use of their unique experiential expertise (Mahlke et al. 2017; Oborn et al. 2019; Yeung et al. 2020) and specific skills gained in their training. The approach arose among the mental health service user movement in the 1990s (Davidson et al. 2012). Since then, the development of peer support work and the implementation in the mental healthcare system has made progress in many countries due to changes in healthcare policies towards patient-centeredness. These changes were led mainly by Anglo-Saxon nations, such as England, Wales, Scotland, New Zealand, Australia, Canada and some states in the USA (Shepherd et al. 2008). Since 2005, PSWs have been educated according to the “Experienced Involvement” curriculum in Germany and other European countries, which is based on the so-called trialog movement (Amering 2010). The one-year education program is currently offered at more than 30 locations in Germany (EX-IN Deutschland 2020), which leads to the fact that more and more PSWs are seeking employment in mental healthcare institutions. There is still a lack of research about the integration and role-finding process of PSWs in psychiatric hospitals as peer support work is a comparatively new profession, at least in Germany.

The Regional Association of Westphalia-Lippe (LWL) initiated the project “Employment and payment of educated PSWs in the LWL Psychiatry Network” in 2011 within the framework of the implementation of the United Nations Convention on the Rights of Persons with Disabilities. The goal of the initiative was to promote the employment of PSWs in adult psychiatric hospitals of the LWL Psychiatry Network. Until that time, the adult psychiatric hospitals participating in this study only had a few experiences with and rare knowledge about peer support work.

Against this background, our research aimed to explore the integration and role-finding process of PSWs into mental healthcare teams in psychiatric hospitals and identify promoting factors. Furthermore, we aimed to clarify how PSWs perceive their roles and how the latter evolve over time.

Up to now, various international studies have already explored the topic of the implementation of peer support work in psychiatry (Chinman et al. 2010; Hamilton et al. 2015; Kent 2019; Siantz et al. 2016). Among these, there were both quantitative and qualitative empirical studies. In most cases, the latter were based on the evaluation of qualitative interviews and focus groups.

To the best of our knowledge, our study is the only one which gathers knowledge by open nonparticipant observation of two settings in adult psychiatric hospitals over a longer period of time. Our longitudinal qualitative observation study was part of a larger project which consisted of three parts (Gather et al. 2019): (1) the observation study presented in this article, (2) qualitative interviews with PSWs, and (3) focus groups with PSWs and mental health professionals. Results from the interviews and focus groups have already been published elsewhere (Otte et al. 2020a, 2020b).

## Methods

### Nonparticipant Observations

We conducted open nonparticipant observations of 25 multiprofessional team meetings (TMs) including one PSW each in wards of three adult psychiatric hospitals of the LWL Psychiatry Network in North Rhine-Westphalia, Germany, from April to October 2016. Additionally, we observed five transregional PSW meetings (PSWMs), i.e. meetings in which the PSWs of the different psychiatric hospitals gathered and discussed their experiences among themselves. Three further PSWs working in two other LWL adult psychiatric hospitals took part in these meetings (see Table 1).

**Table 1:** Overview of the participation of peer support workers (PSWs) in different observation settings

Hospital	Peer support worker (PSW)	Subject to observation during team meeting (TM)	Subject to observation during transregional peer support worker meeting (PSWM)^b^
1	PSW1	Yes, TM1 1–TM1 5^a^	Yes
2	PSW2	Yes, TM2 1–TM2 5	Yes
2	PSW3	Yes, TM3 1–TM3 5	Yes
3	PSW4	Yes, TM4 1–TM4 5	Yes
3	PSW5	Yes, TM5 1–TM5 5	Yes
4	PSW6	No	Yes
4	PSW7	No	Yes
5	PSW8	No	Yes

^a^TM1 5, for example, means: observation of PSW1, fifth team meeting

^b^There were five transregional PSW meetings. We refer to them in our paper as PSWM 1–5

The study has been approved by the Research Ethics Committee of the Medical Faculty of the Ruhr University Bochum (Reg. No. 15-5387). The observations were performed by two researchers with different professional backgrounds (A.N.: Philosophy with a particular focus on medical ethics; A.W.: Molecular biomedicine and peer support work). They were written down on-site in handwritten notes which were then used for immediate documentation using a word-processing program. While creating the documentation, we anonymized all data to make sure that no person observed could be identified. We structured each observation following an observation protocol. We included general data (date, time, people present) and a description of the setting (e.g. the rooms, the positioning of people and the atmosphere) in each transcript of a meeting observed. We depicted the social interactions observed as descriptively as possible. Initial interpretations and thoughts were noted in a separate way to distinguish them clearly from the social practice observed directly. All quotations in this paper are extracts from the observation transcripts. We inevitably came into contact with patient information while observing TMs in wards of psychiatric hospitals. We never documented any patient data since this information was not relevant for our research. Nevertheless, before the meeting took place, all patients who were supposed to be discussed on this TM were informed and asked for their consent. When patients refused or could not be asked, the observers left the room during the discussion of their cases.

### Data Analysis

We analyzed the data in line with the basic principles of qualitative content analysis (Mayring 2014) using MAXQDA (MAXQDA 12 Standard Portable, VERBI Software GmbH, Berlin, Germany), a software for qualitative data management. At first, each setting was treated separately in the analysis. Both first authors analyzed and coded the observation transcripts independently using an open-coding process. They discussed their interpretations and conceptualizations of the data with the other members of the research group, each having a different disciplinary background (sociology, psychiatry, medical ethics, philosophy) until a shared understanding was reached. After that, initial categories capturing the core themes of the TMs and transregional PSWMs observed were developed and the codes were grouped accordingly by A.N. and A.W. The categories were discussed within the research team and then further developed to more central categories.

## Results

There were significant differences between the two settings observed leading to diverging results we could gather in each setting. During the TMs, we were able to observe the direct communication, interaction and behavior of the team including the PSW. Thus, the TMs were the setting most suitable for findings concerning the central category “integration into the team.” In addition, observation of the transregional PSWMs offered the opportunity to learn about the thoughts, feelings and variety of challenges the PSWs faced during their job routine, as those meetings were used predominantly as a space for mutual exchange among the PSWs and had the character of a peer consultation. The central category “PSWs’ roles” was developed mainly out of this setting. All categories, major subcategories and examples are listed in Table 2.

**Table 2:** Central categories, subcategories and examples

Central categories	Subcategories	Examples
Integration into the team	Features of success	Equal treatment in team conversations PSWs have specific knowledge/abilities
	Challenges	Lack of resources Missing contact with mentor
	Positioning between team and patients	Acceptance of being informally addressed by patients Problem of how to pass on patients’ concerns to the staff
PSWs’ roles	Offers	Being available, individual conversations Follow-up care Groups: Recovery, social skills training, creative writing, etc.
	Self-perception	Ambivalence in the judgment of leeway Change from feeling superfluous to gaining self-confidence Support by PSWM and external supervisor

### Integration into the Team

#### Features of Success

Although there are factual inequalities between PSWs and other mental health professionals regarding pay and power, we did not observe any kind of inequality between PSWs and other team members during their communication in the multiprofessional TMs. The respective senior psychiatrist or head nurses appeared to appreciate the contributions of PSWs in general.

Senior psychiatrist to PSW5: “You wanted to say something? You inhaled!” (TM5 2).^1^

The PSWs’ contributions had the same (high or low) significance as those of other team members. Whether or not a PSW contributed often appeared to be connected, first and foremost, with the individual personality of the specific PSW. The PSWs were on a first-name basis with other team members, such as psychiatrists and nurses, and included in the informal chatting (e.g. jokes or small talk) before and after a TM.

The PSWs provided a lot of relevant information about patients during the observation period.

The senior psychiatrist and the psychologist talk about a patient. They comment on the patient’s lack of understanding of their condition and the deficits in behavior to improve their situation.

PSW3: “But they came to me for information about self-help groups!”.

Senior psychiatrist (astonished): “Really?” (TM3 3).

PSW2: “I can give a lot of helpful additional information, things the nurses don’t even notice.” (PSWM 2).

On the other hand, team members asked PSWs to talk to specific patients, include them in a group or accompany them while doing certain tasks.

The psychologist of the ward was sure that one of their patients would benefit from talking to PSW1 and asked her to initiate the contact:

Psychologist to PSW1: “I’d like you to start with patient X immediately!” (TM1 3).

Senior psychiatrist to PSW3: “You’re practicing bus driving with [the patient]?”.

PSW3: “No, but I can do that if there’s a need!” (TM3 2).

The PSWs felt accepted when there was a contact person and this person had time for conversations.

PSW4: “PSW5 and I meet with the head nurse every two weeks.” (PSWM 1).

Additionally, it was relevant that there was ongoing communication with superiors and colleagues to clarify mutual expectations and needs and, moreover, to provide feedback.

PSW1: “I’ve got positive feedback about my presence in groups, because I bring in the patients’ perspective.”

PSW2: “Yes, it was the same for me.” (PSWM 1).

PSW2 stated that she received official feedback after four weeks of working in the hospital. PSW2 “liked that a lot” (PSWM 1).

#### Challenges

We could observe good contact between the PSWs and their contact people in the TMs; however, the relationship was sometimes challenged by a full work schedule. The PSWs also used the PSWMs to talk about their contact people on the ward:

PSW4: “The head nurse has a lot on her mind; you don’t want to rob her of the rest of her free time. That builds up pressure on you.” (PSWM 2).

PSW5: “I guess judging by the [head nurse’s] facial expression that they think: ‘My God, what does [PSW 5] want now?’” (PSWM 2).

On the sidelines of the observations, one PSW told us directly about his problems with the lack of access to resources:

PSW2: “I have suggested the discussion with contact person X. I am tired of the fact that I still haven’t got a staff mail account. Things aren’t running smoothly for me yet!” (TM2 1).

#### Positioning Between Team and Patients

The PSWs expressed their challenges in the PSWMs and the TMs regarding positioning themselves between team and patients. The topics were mainly passing on important patient information, such as suicidal ideation, or conveying personal criticism to the staff. The PSWs experienced themselves not only as part of the team but also relatively close to the patients. This posed an intricacy to the PSWs:

PSW5: “I have a problem personally of conveying criticism to the team. I have to improve [i.e. working on finding the courage to criticize the team].”

PSW1: “You haven’t been present. Maybe you can support the patient better by helping them to report their criticism themselves.” (PSWM 3).

In accordance with the proximity to the patients’ perspective, the PSWs mainly appreciated the case that patients address them informally:

PSW1: “You are well received while making the rounds when the patient forgets to address you formally; I view that as a compliment.” (PSWM 1).

Most of the PSWs did not mind being addressed informally but wanted to emphasize that they are members of the team by still addressing patients formally.

PSW4, for instance, regards it as uncomfortable to address patients informally: “The patients can address me informally, but I keep it formal myself.” (PSWM 3).

### PSWs’ Roles

#### Offers

The PSWs performed a variety of tasks and provided support for patients on different levels during the observation period. The PSWs generally provided one-on-one conversations with patients, attended TMs, took part in ward rounds when asked by patients and did informal tasks, such as attending supper, aiding patients with their kitchen duties and joining patients in going for a walk or running errands. Moreover, providing a bit of normality by offering small talk and just being there, being available for patients was one of the most relevant tasks. Even though the respective activities persisted during the whole observation period, the PSWs talked about their everyday attending offers especially in the beginning.

PSW5: “With one patient I talked about the weather, with the other about her hairdo … but you are available and helpful for their well-being and overall atmosphere.” PSW1: “When I was a patient, I enjoyed small talk and the distraction caused by it.” PSW5: “I can give normality, time. Sometimes I just go and sit down with a woman who is knitting.” (PSWM 1).

Additionally, the PSWs were involved in the follow-up care of discharged patients. The PSWs talked about options for follow-up care repeatedly during the PSWMs. The group, for instance, was very interested in hearing about PSW2, who encouraged the patients to write letters including wishes and goals addressed to themselves, collected them and sent those letters to the patients subsequently an agreed time after discharge (PSWM 5).

Another big part of the work was to develop and host therapeutic groups, such as recovery groups, social skills training or creative writing groups. Regarding such groups, the PSWs worked partly together with other staff members.

PSW1: “I regard the leading of groups as one of my strong suits.” (PSWM 1).

#### Self-perception

The PSWs often expressed the comprehension of their role figuratively, which becomes apparent in several terms and metaphors they used in the PSWMs:

PSW5: “I see myself as a ‘source of hope’.” (PSWM 1).

PSW3: “Patients want me as an ‘advocate’.” (PSWM 1).

PSW6: “My colleagues often call me a ‘pioneer’.” (PSWM 3).

PSW1: “I get involved when, for example, they want to change therapists or when they don’t want to be discharged yet, when the patients have the feeling that ‘I am fighting a losing battle.’ But the patients manage it autonomously, presenting their concerns; I am the ‘backup’.” (PSWM 3).

However, the beginning of the practical role-finding process was difficult for the PSWs:

PSW5: “The head nurse says: ‘No, *you* have to have the suggestions, the ideas!’ I would have liked clear instructions sometimes.”

PSW4: “However, in retrospect, I appreciate these liberties, that nothing was imposed on me. Nevertheless, in the beginning, it [the leeway] was pressure and insecurity.” (PSWM 2).

The PSWs often stated in the PSWMs how important those meetings are for exchange, mutual confirmation and, therefore, role-finding:

PSW1: “I feel like I’m ‘floating’ [i.e. feeling utterly insecure]; I am glad that we have the group.” (PSWM 1).

PSW5: “The meeting has encouraged me!” (PSWM 1).

After the first three PSWMs, the PSWs had the feeling that they would benefit from a trained external supervisor. Therefore, beginning with PSWM 4, each PSWM included a timely limited session with an external supervisor. Respecting the PSWs’ wishes, we did not observe these parts of the PSWMs 4 and 5.

PSW1 enumerates the positive features: “[…] satisfaction with the supervisor, this should be continued. And further peer support worker meetings.” (PSWM 5).

The feared or actual problem of being not able to find access to patients was a recurring issue in the PSWMs.

PSW1: “During lunchtime you encouraged me. In the current phase, when the patients apparently don’t need me so much, I feel superfluous …” (PSWM 1).

Moreover, the PSWs discussed the fact that, considering the number of patients on a ward, it is not possible to have a close contact to everybody. PSW5, for instance, stated that they have learned that “it is not possible to know everyone [i.e. every patient]” (PSWM 1).

In general, we observed that the topics changed in the PSWMs during the observation period. At the beginning, the PSWs talked mainly about their everyday attending offers and their role-finding process. Later, both in the PSWMs and TMs, the focus was more on group offers whereat the PSWs showed assertiveness:

The team talks about what to do best to help a patient become more active. PSW5: “I also integrated the patient into my recovery group.” The other team members murmur approvingly. (TM5 1).

PSW4: “I speak quite frankly in the recovery group, personal stories as well; this goes down well with the patients.” (PSWM 5).

PSW6: “One last thing, do you have a folder for the patients to take away?”.

PSW4: “I give them worksheets.”

PSW1: “That would be a good idea for the recovery group! Making one’s own folder and designing it beautifully!” (PSWM 5).

## Discussion

Our observations revealed that the members of the multiprofessional mental healthcare teams in the psychiatric hospitals appreciated the PSWs’ statements during the TMs. Psychiatrists and psychologists assigned PSWs to specific patients and asked them to include patients in their group offers. This suggests that the mental health professionals understood the specific skills and knowledge of PSWs and were inclined to use the PSWs’ special expertise for the benefit of the patients.

At the beginning of their integration process, the PSWs were concerned about the lack of clarity of their role. This phenomenon of vague roles is discussed in several international publications (Crane et al. 2016; Gates and Akabas 2007; Gillard et al. 2014; Hurley et al. 2018; Ibrahim et al. 2020; Jenkins et al. 2018; Mahlke et al. 2014; Mancini 2018; Miyamoto and Sono 2012; Mowbray et al. 1996; Simpson et al. 2018; Tse et al. 2017; Vandewalle et al. 2016). A qualitative study in the USA (Cabral et al. 2014) and two qualitative studies in the UK (Gillard et al. 2013, 2015a) suggest that this unclarity is a hindrance to the integration of PSWs. However, our observations revealed that PSWs became more secure in their own understanding of their role over time. The vagueness which initially caused insecurity and pressure was appreciated as freedom to design one’s own role definition later on. Asad and Chreim (2016) come to a similar conclusion by stating that some ambiguity can be seen as a benefit for PSWs. The question how much clarification or even standardization of the role is necessary, on the one hand, and how much freedom for individual role-finding is required, on the other hand, is a topic in several publications. Moll et al. (2009) discuss the positive implications of a variable and evolving role and the need for some clarity for PSWs and staff. Heumann et al. (2019), who discuss this issue in detail, are aware of the downside of a fixed role description but still opt for a clear definition of the role. Gruhl et al. (2016), however, conclude that any training or standardization of the role should be based on the authenticity of PSWs, which is to be seen as the core of peer support work. Additionally, the fact that peer support work is a relatively new profession and, therefore, PSWs inevitably lack a basic common identity so far should be considered. Hurley et al. (2018) point this out by comparing the current role-finding of PSWs to the role formation process of mental health nurses.

Based on the data gathered during our observations, we doubt that the PSWs’ different tasks and offers can be grasped in a completely fixed role description in advance. By limiting choices, a fixed role description would undermine the specific way of support PSWs are able to offer. However, a kind of framework role description should be issued in advance to give some guidance to the PSWs. Such a framework gives other mental health professionals an idea about the role of their new colleagues and can, thus, avoid problems during the integration process (Asad and Chreim 2016). This view is in accordance with our own findings in the qualitative interview and focus group parts of our study (Otte et al. 2020a, 2020b) and with the findings of Collins et al. (2016). The latter interviewed psychiatrists about their attitudes towards PSWs in the UK and stated that, given the influence of psychiatrists on other team members, a lack of knowledge about the role of PSWs can pose a danger to their integration.

Gillard et al. (2015b) suggest the local creation of role understandings within a certain mental healthcare team to promote integration. In their view, such an approach can help to reduce the conflict between role specifications that are either too narrow or too vague. Our findings support this idea. All PSWs who participated in our study developed their roles on a local level in relation to the needs of the patients and non-peer staff on their specific wards.

Furthermore, our findings suggest that PSWs benefit from the mutual exchange in transregional PSWMs during their role-finding process. This is in line with the results of our interview study, in which PSWs stated that they perceived these transregional meetings, which can be understood as a peer support for PSWs, as especially helpful (Otte et al. 2020b). Potential strains on PSWs and the need for support on various levels is addressed in several publications (Ahmed et al. 2015; Byrne et al. 2019; Cabral et al. 2014; Davidson et al. 2012; Nestor and Galletly 2008; Simpson et al. 2014). There is evidence that the exchange of experiences and the mutual support is important when implementing peer support in mental healthcare. Davidson et al. (2012), for instance, suggest hiring at least two PSWs for any ward or team, respectively. Our findings support Davidson’s claim. In our study, PSWs were not employed in tandem, however, they experienced it as relieving to know that they are not alone and that other PSWs have similar questions or problems. Additionally, they could give each other advice if needed. The fact that PSWMs generate a safe space in which PSWs can talk openly without having the fear that they could unintentionally offend other mental health professionals seems very important.

Moreover, our findings suggest that regular communication with mentors on the wards and other team members is both helpful for the development of a professional role of PSWs and crucial for their integration into the multiprofessional team. These results are in line with Debyser et al. (2019), who recommend that care providers should establish a support framework for PSWs in collaboration with the PSWs themselves. Davidson et al. (2012) further suggest giving an administrator the role of a “peer staff ‘champion’” (p. 127), who is supposed to intervene when problems on an organizational level occur. We also think that—regardless of what it is called or how concretely it is organized on a local level—the importance of strong communication and support structures for PSWs and other team members cannot be stressed enough.

### Strengths and Limitations

To the best of our knowledge, our study is the first longitudinal qualitative observation study which gathers knowledge on the integration and role-finding process of PSWs in adult psychiatric hospitals in Germany. Observational studies are rare, which might, at least partially, have to do with the time and effort they need. Observations can give insights into certain routine actions which often cannot be obtained by other qualitative methods as they are unlikely to be mentioned in an interview or a focus group (Harvey 2018). Moreover, observations show the social context in which people communicate and act (Salmon 2015). Another advantage of observational studies is that they enable the researcher to circumvent the social desirability bias by showing not only what people say but also how they act (Mays and Pope 1995). Although people may attempt to present themselves in the best light, it is not possible to do so over a longer period of time, especially once they get used to the presence of an observer (Mulhall 2003).

In addition to these strengths, there are also limitations which are specific to an observation study. An observation changes the situation observed in two ways. Firstly, any observation influences the social behavior observed. Such influence is unavoidable. Therefore, we tried to limit our impact by asking all the participants to act as they usually do and by acting with particular reserve. Secondly, any observation is shaped by the perception of the observer (Mulhall 2003; Salmon 2015). Therefore, it is necessary to reflect thoroughly and to discuss initial interpretations within the research team. Furthermore, we added remarks about potential subjective factors which might influence the perception of the practices observed during the analysis. The different professional backgrounds of the observers and research group members were also helpful and important to avoid methodical biases, such as over-identification.

Furthermore, qualitative-empirical research has some general limitations. Our study is based on a limited number of observations in a specific type of adult psychiatric hospital in Germany. Considering the differences between hospitals on an organizational level, especially in an international context, and the influence of the individual personalities of the people involved, it is not possible to generalize our results.

## Conclusion

Integration of PSWs into multiprofessional mental healthcare teams in adult psychiatric hospitals is possible and mental health professionals appreciate the special expertise of PSWs. The PSWs’ specific roles evolve in a process over a longer period of time and aligned with the specific needs on a local level. This role-finding process should be supported by a framework role description, on the one hand, and sufficient freedom for individual development, on the other hand. Regular opportunities for mutual exchange, such as the offer of “peer support for PSWs” at transregional PSWMs, can help to promote the evolvement of roles and to address specific support needs at different points in time.

## Footnotes

1 The senior psychiatrist addressed the PSW informally. In German, one says “Du” instead of the formal address “Sie”.
